# A qualitative study of employees’ opinions on establishing a generic call-centre

**DOI:** 10.1186/s12875-017-0661-x

**Published:** 2017-10-17

**Authors:** Hilde Carin Storhaug, Sara Bjune Mead, Aslak Steinsbekk

**Affiliations:** 1 Health and Welfare Services, City of Trondheim, Norway; 2 Department of Public Health and General Practice, Norwegian University of Science and Technology, Trondheim, Norway

**Keywords:** Call-centre, Telephone triage, Out-of-hours general practitioner service, Safety alarm service, Primary health care

## Abstract

**Background:**

To investigate opinions among employees at an Out-of-Hours general practitioner (OOH-GP) service and a safety alarm service about the establishment of a generic call-centre.

**Methods:**

Qualitative study using individual and group interviews with 14 employees and managers involved in preparation of a merge into a new generic call-centre. They were asked about their opinions towards establishing a generic call-centre where all contact about unplanned health inquiries from the public had to be done by telephone and how to solve more requests on the phone. Data was analysed thematically.

**Results:**

Participants who alternate between call handling and direct patient contact (personnel at the OOH-GP) believed that just handling calls would be monotonous, less challenging and provide poorer quality. This was not supported by those working at the safety alarm service. There were different opinions about introducing mandatory use of decision support system for all inquiries, but it was a common understanding that it would lead to more patients in need of face-to-face consultations due to over triage. To solve more requests on the phone participants believed a public information campaign was required, that GPs received more of the emergency requests within their ordinary working hours and having salaried doctors in the OOH-GP service.

**Conclusion:**

In the participants’ opinion, successful establishment of a generic call-centre depends on the employees’ possibility of direct patient contact, clarifications on the use of decision support system and good information to the population.

## Background

To try to reduce the number of Out-of-Hours general practitioner (OOH-GP) services and unplanned hospital admissions, call-centres and call-based triage sorting has become more common [1, 2]. Organizational structure and objectives varies from different call-centres [3], and there is also substantial variance in the type and urgency of enquiries the call-centre employees must handle, including infections, neurological, cardiac, soft tissue and mental health issues [4].

Call-centre work has been described as an information technology-supported, communication-intensive form of work [5]. Research has shown that call-centre employees often experience high work intensity and limited influence over own work schedule [6]. They often have to work unusual hours and there is also a high rate of part-time employment [5]. A study conducted among nurses working at a Swedish telephone advisory service found that nurses described being both a gatekeeper and a carer as mentally demanding [7]. However, it has been found that having more complex tasks was associated with experiencing lower stress levels and better psychosocial conditions in call-centre workers [8].

Decision support systems are increasingly being used in call-centres to support the decision-making process [9]. Such systems are used to facilitate and ensure quality in the process of determining the priority of patients’ treatment needs based on the severity of their condition (triage). It has been found that using a triage-sorting system can reduce visits to the Out-of-Hours GP services [10]. However, it has also been found that they can both impede and facilitate the decision-making process [11], and might be experienced as controlling by the call-centre operators [12].

Merging different call-centres, like Out-of-Hours GP service and safety alarm service, to one could make it easier for the population to know where to call in case of emergency [13]. Although there are some publications on the experience of working in generic call-centres that handles different types of contacts [7, 14, 15], we have not found any study on the opinion of call-centre operators who are to be merged into a generic call-centre. Such knowledge can be used to improve the process of merging call-centres.

The objective of this study was therefore to explore the opinions of employees at an Out-of-Hours general practitioner service and a safety alarm service about a forthcoming merge into one generic call-centre for all unplanned health inquiries.

## Method

This was a qualitative study with semi structured individual- and focus group interviews. The data collection took place between September 2013 and February 2014. All participants received and signed written information about the study objectives, how the data would be handled and that participation was voluntary. As the project did not concern health research on the participants, approval by the Regional Committees for Medical and Health Research Ethics was not necessary. The Norwegian Social Science Data Service approved the study (case number: 27,657).

### Setting

Four municipalities and one city in Central-Norway have been collaborating on establishing a new model for municipal 24-h emergency services. They are establishing a call-centre that will receive all inquiries and secure appropriate follow-up of the enquiries that cannot be solved over telephone (Table 1). Two units are affected, an inter-municipal Out-of-Hours GP (OOH-GP) and a municipal safety-alarm service.

**Table 1:** Information about the generic call-centre

The generic call-centre will be the point of a contact for all unplanned health inquires for the population in the involved municipalities. Based on the judgment of the call handlers, the patients will get their problems solved over the phone, they will get an appointment with the GP at the stationary Out-of-Hours GP service or receive a visit from the ambulant GP service or safety alarm.
It will be staffed 24/7 with competent employees, using standardized decision support systems to ensure uniform handling of all inquiries.
It is estimated that 80% of the inquiries can be solved on the telephone, resulting in a reduction of face-to-face GP consultations and unplanned hospital admissions.

The OOH-GP provides service to a population of 220,000 inhabitants and is located close to the emergency unit at the university hospital in the city. In addition to an ambulant unit, it is a call-centre co-located with the stationary unit where the nurses shift between handling phone-calls, receiving patients and coordinating the clinical activity.

The safety alarm service (a personal emergency response system) comprises an ambulant unit and an alarm call-centre receiving and responding to safety alarms from approximately 4000 inhabitants. The safety alarm is a device that is worn as a bracelet with a button to press to get contact with the call-centre. It is provided to persons who feel unsafe when living alone. The employees, who do not need to have a nursing background, work either in the ambulant unit, or at the call-centre. All the safety-alarm employees have experience with handling OOH-GP contacts from prior employment at a private emergency call-centre.

### Participants

The aim was to include participants from the 2 units; leaders with personnel responsibility, employees in the most central roles in the merge project and employees considered to be potential call-centre employees. Variance in employment status, gender and number of years in current employment was considered when choosing participants.

Potential participants were sent an email with invitation to an interview. These were either known by the researchers through previous informal observational activity in the project, or employees recommended by leaders or other participants when they were asked for participants who had taken part in the different activities or who had specific roles at work. Those willing to participate contacted the study and got an appointment for the interview with the first author (HCS).

### Data collection

Data was collected through semi structured individual and focus group interviews. Focus groups were preferred for employees without leader responsibility, as the interaction in a group can yield associations and reflection on each other’s opinions [16]. To avoid that participants with leader responsibilities would affect the dynamic in the focus group interviews with the staff, the leaders were interviewed individually. The interviews, all conducted by the first author (HCS) at the participant’s work place during normal working hours, were introduced with some information about the plans for the call-centre (Box 1) and the objective of the study. The interviews were audio recorded and lasted from 45 to 90 min, with an average duration of 65 min for the focus group interviews and 54 min for the individual interviews.

To give the researchers a better understanding of the context and the merge process, informal conversations, observation of different types of meetings and site visits were conducted during the data collection period. These data were not included in the analysis directly, but used to understand the responses in the interviews.

The interview guide was developed by the authors, based on their knowledge of the project, research literature on experiences with working in call-centres and discussions with central people in the project. The main questions relevant for this article was “what are your thoughts about handling all unplanned health inquires through telephone or technology in the call-centre?” (Table 2).

**Table 2:** Interview guide. Topics not relevant for this article is removed. The emphasis on and order of the questions was adjusted somewhat during the interviews

Introduction given to the informants
- Short information about the generic call-centre’s future role as an independent unit and the aim of this study.
- Make sure the participants have received the cover letter and consent to participate
About the participant
- Work-place, job title, percentage of employment, time in current position, education, other relevant practice.
Main questions
What are your thoughts about handling all unplanned health inquires through telephone or technology in the call-centre? (ask follow up questions and get the informants to talk as freely as possible about the main question)
- What are your thoughts about working in a generic call-centre?
- What challenges and opportunities do you see?
- What do you think about having direct patients contact or not?
- How do you look on call-centre work? (Monotonous or straining work?)
- What are your thoughts on having GPs present or absent in the premises?
- Do you think of the work as professionally challenging/boring/unsafe/stressful?
- What will make the call-centre an attractive work place?
- What is decisive if you decide to work there?
- If you don’t want to work at the call-centre, why is that?
Specific questions
What do you think about the information you have got about the background and cause for choosing the new model?
The plan is that nurses only will operate the generic call-centre. What are your thoughts on that?
What do you think about mandatory use of a decision support system
Do you think that it is likely to reduce the number of face-to-face consultations by solving more requests over the phone?

### Analysis

The interviews were successively transcribed, summarized and analysed thematically with ad hoc opinion generation and analysing techniques described by Miles & Huberman [17, 18]. HCS did the initial analysis and discussed it with AS who read about half of the transcripts. Then SM read all the transcripts and suggested adjustments. To get a general impression of the participant’s opinions, topics directly linked to the research question were first written down. The interviews were subsequently studied with an aim of identifying patterns and themes before a general summary was made. Themes identified in the first round were compared to themes identified in the second round. In the further analysis, the themes were readjusted and closely assessed for potential differences between employees at the two different call-centres and between leaders and employees. The opinions varied substantially between employees at the two different units, but within each unit the employees and leaders had quite similar opinions. Therefore, the differences between each unit is highlighted in the results. The analysis was continuously discussed between the authors and all the original text was re-evaluated. People involved in the project were informed about the results, and confirmed that they were consistent with their impression.

## Results

In total 14 persons were interviewed, seven individually and seven in two separate focus groups (Table 3). Seven participants came from the Out-of-Hours general practitioner (OOH-GP) service and seven from the safety alarm service. About half (8 out of 14) of the participants had experience from working as call handlers at a OOH-GP call-centre. Most (12 out of 14) had worked in their current position for over 3 years and a half of the participants worked fulltime.

**Table 3:** Characteristics of the participants (*N* = 14)

Characteristics	*N*
Employed at	
OOH-GP	7
Safety-alarm services	7
Gender	
Female	9
Male	5
Education	
Vocational health education	6
Registered nurse/ welfare nurse	5
General practitioner	3
Role	
Leader (with personnel responsibilities)	3
Professional leader/Professional Developer/management responsible	3
Employee	8
Experience from current employment	
0–3 years	2
Over 3 years	12
Employment form	
Part time	7
Full time	7
Working hours	
Day time only	4
Rotation	10

It was evident that the opinions were influenced by the participant’s current workplace. In general, participants from the safety-alarm central were more positive to establishing a generic call-centre, while the participants from the OOH-GP were more sceptical and reluctant. The findings were categorized into three main themes; Challenges with a generic call-centre, Decision support system, mandatory use or not? and How to solve more inquiries over the telephone?

### Challenges with a generic call-centre

Most of the participants were familiar with the main idea of the call-centre. Even though there were only a few participants who thought the whole idea behind a generic call-centre was fundamentally wrong, several of the employees talked about important challenges that made them consider the merge as a bad idea. Some participants from the OOH-GP were concerned that their tasks in the call-centre would be monotonous and less professionally challenging, as it would consist of “*only answering the telephone”*. They also said that the work at the call-centre would be overwhelming, as the same person would have to handle different types of inquires *“…we have to be oracles to handle the call-centre”.* Neither of these issues were expressed as a problem by employees at the safety-alarm central, as they were used to only working with call handling. Additionally, the employees at the safety-alarm central suggested that the operators could have responsibility for administrative and management tasks, like processing statistics and testing equipment, in addition to handling the calls.

Some employees from both the OOH-GP and the safety alarm central expected the call-centre to be a new and inspiring place, and highlighted that a generic call-centre would give a larger work environment with more competence united at one place.

*“We can hope that the call-centre will be the pulsating vibrant heart of the services for unplanned health inquiries, filled with competent workers, - a place people really want to work at.”* (Safety alarm).

With the plan to make the call-centre a gatekeeper for access to the OOH-GP and ambulant alarm service (Box 1), all inquiries from the public should be made by telephone or other forms of technology. The participants from the safety alarm believed that when people got used to calling first, it was realistic to expect fewer face-to-face contacts as their experience was that they could solve many problems over the phone. The participants from the OOH-GP did not expect this result.

“*The population will never accept that they have to call first. Patients will still show up unannounced, and it is way harder to reject them at the counter than on the telephone.”* (OOH-GP).

Many of OOH-GP employees said that the quality would deteriorate if the call handlers did not also work with face-to-face clinical assessments of the patients or were not able to consult with a doctor directly due to the call-centre being physically separated from the stationary unit. They therefore voiced strong opinions that the call-centre must be organized in a manner that gave them an opportunity to alternate between call handling and direct patient contact in the stationary unit.

*“To have seen the patients that are treated at the OOH-GP for yourself is crucial to handling the calls correctly”.* (OOH-GP).

### Decision support system, mandatory use or not?

The plan to use an evidence- and algorithm based decision support system for emergency assessment (triage) was met with both scepticism and applause. Some participants from the OOH-GP were sceptical to mandatory use as it would be a barrier for basing decisions on consultations with doctors, their own experiences and professional assessment. They suggested that it should be a supplement, but that it could be mandatory for inexperienced operators. There were others that argued strongly for making the system mandatory to avoid what was termed not-evidence based decisions.

*“… all work is centred around competence and quality; one is not here to base advice on one’s own opinions”* (OOH-GP).

There were diverging opinions among the safety alarm employees on whether the safety alarms also should be handled through the decision support system. Those arguing for, said that they had experienced that patients with safety alarms often used this in case of emergency instead of making an ordinary call, and that the system therefore also should be applied to safety alarms.

Some of the OOH-GP participants believed that mandatory use of decision support systems would increase the number of patients in need of a face-to-face GP assessment as many callers exaggerate their symptoms, leading to over-triage. Others argued that patient and operator safety was the most important, and when in doubt the patient should always be assessed by a GP and said that over-triage should be considered a quality assurance.

*“I fear that there will be more yellow responses with mandatory use, it will increase the number of visits due to how the system is built. Today’s system is good, don’t know about any reasons for why the new system should be used” (OOH-GP).*

### How to solve more inquiries over the telephone?

To solve more inquires over the telephone and thus reduce face-to-face consultations, some believed it was necessary with a widespread information campaign to educate the population to call before turning up, and that such a campaign should start in good time prior to the implementation.

*“It depends on how you in advance inform the public, which is not done with one ad in the newspaper. A massive campaign must start in good time to get a large group to change behaviour. This is very important* [said with strong emphasis] *in making this a success”.* (OOH-GP).

It was pointed out that many patients today physically showed up at the OOH-GP due to not getting through on the telephone. It was therefore said that to solve more inquiries over the telephone, it would be necessary to increase the accessibility by having more call handlers and implement a good queuing system.

Some wanted clearer instructions on which phone calls to reject and when to tell the patient to call their regular GP instead of using the OOH-GP service. Some also said that many do not know that it is their regular GP that is responsible for unplanned health inquiries during office hours. They therefore pointed out a need of making the patients, GPs and operators conscious of this.

*“Very many are not aware that the GPs are responsible for their patients during day-time. We accept too many* [at the OOH-GP]*. The current OOH-GP is a 24-hours open health centre”.* (OOH-GP).

Some also said that as long as the doctors working at the OOH-GP do not have fixed salaries, but rather get paid for each consultation, they have an economic incentive of patients turning up at the OOH-GP. This is due to face-to-face consultations having higher remuneration than telephone consultations. Some participants therefore suggested to permanently employ GPs at the OOH-GP.

## Discussion

The main finding was that the Out-of-Hours general practitioner (OOH-GP) employees who currently alternated between call-centre and direct patient contact were clearly more negative to the idea of establishing a generic call-centre where the call handlers only worked in the call-centre. They thought it would be monotonous and less professionally challenging and that the quality would deteriorate without direct patient contact. There were different opinions about mandatory use of decision support system for all inquiries, but agreement that this could lead to increased number of face-to-face consultations (over-triage). To solve more inquiries over the telephone the participants reported that an information campaign was necessary, that regular GPs should handle the patients currently using the OOH-GP service during regular GPs office hours and that the medical doctors in the OOH-GP should be employed with a fixed salary.

There was a clear difference in the opinions about the establishment of the generic call-centre between employees at the two different call-centres in this study. One likely reason is that those working in the safety alarm service already worked with only call handling, while the OOH-GP employees were most likely to experience the greatest change in their current working situation [19, 20]. Resistance to change might amongst other things originate in a fear of the unknown, loss of identity and breach of a psychological contract; “this is not what I was hired for” [19]. It has been claimed that tele-nursing challenges the traditional role of nursing which encompasses humanistic values, holism and caring as central tenets [21]. It is therefore plausible that the OOH-GP employees also might feel that their identity as a nurse is challenged by only working with call-handling.

### Few professional challenging tasks in call-centres?

The participants who were used to alternate between call handling and direct patient contact (OOH-GP employees) wanted to continue this practice in the generic call-centre to ensure varied and professional challenging work. Similar findings have been reported in previous studies [14, 22], and it has also been found that variety in working tasks is important for job satisfaction in call-centre operators [5]. In Norway alternation between call handling and direct patient contact is recommended [13, 23], but there is a lack of evidence for direct patient contact being decisive for the quality of call handling [24]. As suggested by the safety alarm employees, shifting between call handling and clerical work might provide the sufficient variety [5].

Another aspect is if it is less challenging to only handle calls. Research has found that monotonous tasks might cause job-related strain for call-centre operators [15], while complex tasks are found to enhance working conditions and reduce stress [8, 15]. However, providing medical advice is regarded a complex task as it requires the operators to use their professional knowledge [15]. A Swedish study found that those working full time with call handling found it to be varied, interesting, challenging and competence enhancing as there was something unique about every call and every person [7]. The organizational structure of call-centres can allow nurses to provide care to more people, experience a range of new challenges and might also be less physically demanding than working in a hospital [14, 22].

### Solve more inquires over the telephone

The safety alarm employees believed that the generic call-centre could lead to reduced face-to-face consultations, while the OOH-GP employees were sceptical. Two systematic reviews evaluating telephone triage and consulting services supports the safety-alarm employees’ beliefs; concluding that about half of the calls were managed by phone only, without having to refer the caller elsewhere, and it reduced the number of unplanned visits to the physician without causing an increase in emergency visits [1, 25]. A Swedish study found that attendance at the emergency department was reduced if the call-centre could book an appointment at the patient’s regular GP for the following day, or if the operator could give advice and counselling to inquiries that patients could handle themselves [26].

Some of the participants were sceptical to whether the population would accept that they had to call before getting face-to-face consultations. They suggested that a widespread information campaign prior to implementation of the new system was necessary to educate the population of calling first. A study looking into the implementation of a similar system, found that inadequate information previous to the implementation lead to a phase of low satisfaction in the population, but that the population became more satisfied once they got used to the system [27]. This supports the participants’ call for a comprehensive information campaign, and that it should start in good time prior to the implementation.

It was said by the participants in this study to be a problem that too many use the OOH-GP as if it was a 24-h health care centre, even though the regular GPs are responsible for his/her list-patients’ unplanned inquiries during day-time [28]. It has been found that the OOH-GPs in Norway have different practice on which inquires to accept, some are very restrictive, while others are not [23]. This variation in practice indicates that it is possible to change which types of inquiries that are handled by the OOH-GP. Thus, it is likely that a stricter practice could reduce the use of OOH-GP service during regular GPs office hours as alleged by the participants. However, McGuigan & Watson [4] point out that this would only have marginal effects, as patients who attend the emergency department tend to attend primary health care services frequently too.

### Mandatory use of decision support systems

Some participants argued for mandatory decision support as they thought it would increase quality and equality in how the inquiries were handled, while others thought mandatory use of decision support systems would be a barrier for basing decisions on clinical judgement. Nurses working at NHS Direct in England, which closed in March 2014, expressed the same concerns with decision support systems; making it difficult to exercise decision-making autonomy [11]. Research has found that especially algorithm based decision support systems may infer with the operator’s professional integrity and be experienced as controlling [12]. On the other hand, it has been found that telephone triage is challenging, especially for inexperienced and untrained nurses, and that decision support systems simplifies the work, complements the nurse’s competence and provides a feeling of security [29].

Some participants thought mandatory decision support would lead to more face-to-face consultations as the system would recommend this for patients who exaggerated their symptoms. This concern is supported by other studies that have found over-triage as a consequence of decision support systems [30], and especially when the operator is in doubt about the severity [11]. However, it has been found that mandatory decision support may enhance the patient safety by identifying the patients who understate their symptoms [31].

The strength of this study is that it is the first study to examine employees’ expectations towards establishing a generic call-centre for unplanned health inquiries in primary care. An additional strength is variation in the sample, which also includes medical doctors and employees with previous experiences from different types of call-centre work (Table 2). The main limitation is that the participants were asked about a future situation, and it is therefore unknown how they would experience the situation after working there for a while. The study is conducted in a specific context (Norway) and may therefore have limited transferability. However, other studies have made similar findings [11, 14, 22], indicating that some of the findings are applicable in other settings as well.

## Conclusion

In the participants’ opinion, successful establishment of a generic call-centre depends on the employees’ possibility of direct patient contact, clarifications on the use of decision support system and good information to the public sufficiently early.

## Abbreviations

GP: General practitioner
OOH-GP: Out-of-Hours general practice
